# *Dendrobium officinale* Prevents Early Complications in Streptozotocin-Induced Diabetic Rats

**DOI:** 10.1155/2016/6385850

**Published:** 2016-02-29

**Authors:** Shao-zhen Hou, Chu-yan Liang, Hua-zhen Liu, Dong-mei Zhu, Ya-yun Wu, Jian Liang, Ya Zhao, Jian-ru Guo, Song Huang, Xiao-Ping Lai

**Affiliations:** School of Chinese Pharmaceutical Science, Guangzhou University of Chinese Medicine, Guangzhou Higher Education Mega Center, Guangzhou 510006, China

## Abstract

*Background. Dendrobium officinale* (DO) Kimura et Migo is a precious Chinese herb that is considered beneficial for health due to its antioxidant and antidiabetes properties, and so on. In this research, we try to determine the preventive effect of DO on the early complications of STZ-induced diabetic rats.* Methods*. Type 1 diabetic rats were produced with a single intraperitoneal injection of STZ (50 mg/kg). DO (1 g/kg/day) was then orally administered for 5 weeks. Blood glucose, TC, TG, BUN, CREA, and GSH-PX levels were determined, and electroretinographic activity and hypoalgesia were investigated. Pathological sections of the eyes, hearts, aortas, kidneys, and livers were analyzed.* Results*. Treatment with DO significantly attenuated the serum levels of TC, TG, BUN, and CREA, markedly increased the amplitudes of ERG a- and b-waves and Ops, and reduced the hypoalgesia and histopathological changes of vital organs induced by hyperglycemia. The protective effect of DO in diabetic rats may be associated with its antioxidant activity, as evidenced by the marked increase in the serum level of glutathione peroxidase. However, DO had no significant effect on blood glucose levels and bodyweight of diabetic rats.* Conclusions*. DO supplementation is an effective treatment to prevent STZ-induced diabetic complications.

## 1. Introduction

Partly due to a rapid rise in unhealthy life styles, the number of people with diabetic mellitus (DM) is increasing globally in epidemic proportions. DM can produce devastating effects; for example, hyperglycemia has been found to play a key role in reactive oxygen species (ROS) generated damage [1, 2]. Many scientific reports indicate that diabetic complications are associated with the overproduction of ROS and the accumulation of lipid peroxidation by-products. Diabetic complications can often include but are not limited to nephropathy, neuropathy, and cardiopathy [3–7]. Previously, no effective chemical has been widely used to prevent diabetic complications. The World Health Organization (WHO) recommended the evaluation of traditional plant treatments for diabetes as they are potentially effective with minimal to no side effects [8].

*Dendrobium officinale* (DO) Kimura et Migo is a precious Chinese herb that is considered beneficial for health due to its antioxidant, antidiabetes, immune stimulating, antitumour, and anti-ageing properties [9, 10]. In clinic, DO has been widely consumed as food supplement to enhance body health for hundreds of years. It has also been applied to ameliorate the symptoms of diabetics as it can manifest dry mouth and impaired vision. But there are few reports of the application of DO in amelioration of diabetic complications. In our previous studies, DO exhibited evident antioxidant and anti-inflammatory effects in several animal models induced by oxidants, such as galactose, carbon tetrachloride, and alcohol (unpublished observations). We hypothesised that DO could be effective against the diabetic complications produced from significant oxidative stress and inflammation. The aim of our present study was to investigate the effects of DO on the early complications in streptozotocin-induced diabetic rats.

## 2. Methods

### 2.1. Materials

Kits to measure blood glucose, total cholesterol (TC), triglyceride (TG), urea nitrogen (BUN), and creatinine (CREA) were purchased from the Beijing Zhongsheng Chemical Factory (Beijing, China). Streptozotocin (STZ) was purchased from Sigma Chemicals (St. Louis, MO, USA). The other chemicals used in this study were reagent grade from commercial sources. The kit for quantifying glutathione peroxidase (GSH-PX) was purchased from the Nanjing Jiancheng Chemical Factory (Nanjing, China).

Fresh* Dendrobium officinale* (DO) were provided by Guangdong YongShengYuan Bio-Tech Co., Ltd (batch number 20120110) , which was identified by Professor Lai Xiao-ping in School of Chinese Pharmaceutical Science, Guangzhou University of Chinese Medicine. A voucher specimen is preserved in Guangzhou University of Chinese Medicine. Fresh DO juice was produced using a juice extractor and filtered with gauze. Then, the juice was boiled for 3 min and kept at 4°C until use. The dry stems of DO from the same batch were collected for quality analysis.

### 2.2. Determination of Polysaccharide and Mannose Content

According to the Pharmacopoeia of the People's Republic of China in 2010, the total DO polysaccharide content of the dry herb was assessed by ultraviolet spectrophotometry, and the amount of mannose and the major constituent were analyzed by HPLC. Briefly, 0.3 g powdered stems (350-mesh) were preextracted by 200 mL boiled distilled water in a Soxhlet system for 2 h. The elution was cooled to room temperature and added water to 250 mL. Then 2 mL extraction was mixed with 10 mL alcohol. The mixture was kept in 0°C for 1 h and then centrifuged at 4000 rpm for 20 min. The residues were purified with 80% ethanol twice and finally dissolved in 25 mL hot distilled water. The yield of DOP was determined by the phenol-sulfuric acid method.

Before HPLC analysis, 1-phenyl-3-methyl-5-pyrazolone (PMP) derivative of mannose was prepared. Briefly, 0.12 g powdered stems were preextracted by 80% alcohol in a Soxhlet system for 4 h. The residues were dissolved in 100 mL distilled water. The solution was added 2 mL of 1.2 g/L glucosamine hydrochloride (HAG) and mixture was decocted for 1 h. The volume of the mixture was kept in 100 mL by adding distilled water when cooled to room temperature, and the mixture was centrifuged at 3000 rpm for 5 min. 1 mL supernatant was collected to react with 0.5 mL 3 M hydrochloric acid and kept at 110°C for 1 h under the nitrogen atmosphere. The reaction mixture was cooled to room temperature and centrifuged at 1000 rpm for 5 min. Then the supernatant was neutralized to pH 7.0. 400 *μ*L of the neutralized supernatant was mixed with 0.5 M methanol solution (400 *μ*L) of PMP and 0.3 M sodium hydroxide (400 *μ*L). The mixture was kept at 70°C for 30 min and neutralized after cooling to room temperature. The solution was dissolved in chloroform (2 mL). After vigorous shaking and centrifuging, the organic phase was carefully discarded to remove the excess reagents. The extraction process was repeated three times and the aqueous layer was filtered through a 0.22 *μ*m membrane. The analysis of PMP-labeled mannose was carried out on the RP-HPLC (LC-2010A, SHIMADZU, Japan) on system equipped with a LC-20AT pump, a SIL-20A autosampler, and SPD-20A detector. PMP-labeled mannose was analyzed on an Agilent ODS column (218 mm × 4.6 mm; particle size 5 *μ*m; Agilent Technologies, USA) at 25°C. Running conditions included injection volume, 10 *μ*L; mobile phase, 80% acetonitrile, and 20% ammonium acetate (20 mM), on a gradient run; flow rate, 1.0 mL/min; detection at 250 mm. The quantitative analysis was performed using external standard method. Pure standards of HAG, mannose and glucose were used as internal and external standards to identify the compound. The amount of mannose was expressed as percent of the dry stems of DO.

### 2.3. Animals and Treatment

Male Sprague-Dawley rats were provided by central animal house facility of the Guangzhou University of Chinese Medicine. Rats were acclimatised in an air conditioned room for 7 days (22 ± 2°C with a 12 h light and 12 h dark cycle). All animals were given standard laboratory diet and tap water* ad libitum* before experiment. The studies were approved by the Animal Ethics Committee of Guangzhou University of Chinese Medicine.

Type 1 diabetic rats (280–320 g) were induced with a single intraperitoneal injection of STZ (50 mg/kg). One week later, animals with blood glucose levels >16.7 mM/L were considered diabetic and randomly divided into either the type 1 diabetic control group (DM, *n* = 10) or the diabetic group that orally received 1 g/kg of DO (DM + DO, *n* = 10, this dose exhibited antioxidant and anti-inflammatory effects in our previous studies, unpublished observations). Sex- and age-matched control animals were injected with the citrate buffer vehicle and served as the normal control group (NC, *n* = 8). The diabetic state of each animal was confirmed by evaluation of glycaemic levels every week with a blood glucose meter (Accu-Check Active 1, Roche Pharmaceutical Ltd., Basel, Switzerland). Experiments were performed 5 weeks after the induction of diabetes.

### 2.4. Measuring Mechanical Hyperalgesia

Four weeks after DO treatment, mechanical hyperalgesia in the hind paw of rats was measured using the Electro von Frey method as previously described [11]. Each animal's baseline withdrawal thresholds were determined after the 30 min acclimation period. An automated apparatus (ALMEM-2390-5, Electro von Frey, Life Sciences, Woodland Hills, CA) was used, consisting of a hand-held probe unit connected to a main unit. Five withdrawal readings were taken on the right hind paw of each rat, and the mean of them was used as the withdrawal threshold. The interval between each of the five withdrawal trials was 2 min at least.

### 2.5. Electroretinography

Five weeks after DO treatment, rats were food deprived for 10 hours to assess the electroretinographic activity of each rat with a visual electrophysiological instrument (APS-2000AER, Kanghua Rui Ming Technology Co., Ltd., Chongqing, China). As previously described [12–15], FERG were recorded from both eyes simultaneously during a total of 5 responses to flashes of unattenuated white light (20 ms, 0.05 Hz) from a photic stimulator (light-emitting diodes) set at brightness of 0.006325 cd*∗*s/m^2^ and filtered by a digital band-pass filter from 0.1 to 300 Hz.

The amplitude of a-waves and b-waves of each group were averaged. Oscillatory potentials (OPs) were assessed from 10 responses with an interstimulus interval of 20 ms by filtering the original responses elicited by a stimulus luminance of 0.006325 cd*∗*s/m^2^ by a frequency band-pass filter (50–200 Hz). The sum of four OPs was used for statistical analysis.

### 2.6. Measurement of Blood Biochemical Parameters and Histological Examination

Blood samples were taken under anesthesia by removing the eyeball after testing the electroretinographic activity. The serum samples were obtained by centrifugation (1000 g × 10 min, 4°C) for the measurement of blood biochemical parameters. Blood glucose, TC, TG, BUN, and CREA levels were determined by using commercial kits. All animals were overdosed with pentobarbital sodium (200 mg/kg, i.p.) and immediately sacrificed. Eyes, hearts, kidneys, livers, and aortas were removed promptly and fixed in 10% neutral-buffered formaldehyde for 48 h, embedded in paraffin, and sectioned at 5 *μ*m for histological study. The sections were stained with haematoxylin and eosin and analyzed by masked observers with microscope (IX2-SP. Olympus Corporation, Tokyo, Japan).

### 2.7. Statistical Analysis

All the data were expressed as the mean ± S.E.M. A one-way analysis of variance (ANOVA) test was performed within group comparisons, and a* post hoc* test was performed to determine the significance between different groups. A probability level of less than 5% (*P* < 0.05) was considered significant.

## 3. Results

### 3.1. Analysis of Polysaccharide and Mannose

**Figure 1:**
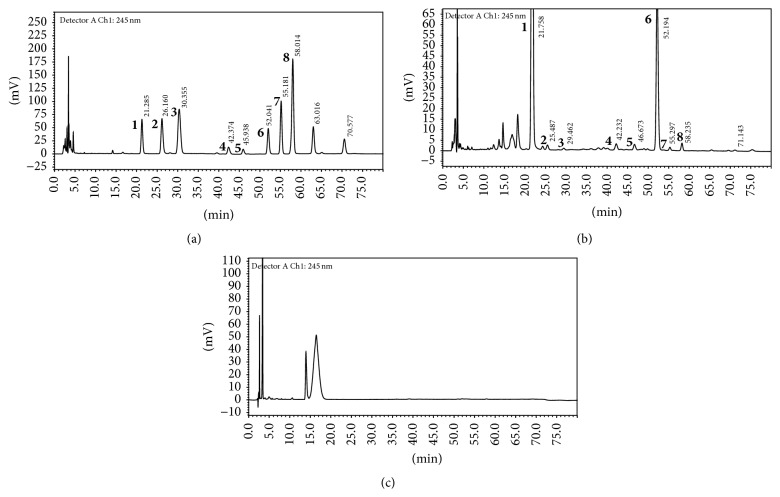
HPLC profile of positive control (a), the polysaccharide fraction of DO (b), and negative control (c). The result indicated that DOP contained several main compounds including mannose (peak 1, 3.054%), ribose (peak 2, 0.030%), rhamnose (peak 3, 0.061%), glucuronic acid (peak 4, 0.028%), galacturonic acid (peak 5, 0.027%), glucose (peak 6, 1.296%), galactose (peak 7, 0.005%), and xylose (peak 8, 0.030%).

Followed by the methods described in the Pharmacopoeia of the People's Republic of China in 2010, the total DO polysaccharide is 30% (w/w) according to the phenol-sulfuric acid method. It can be seen that the PMP-labeled mannose of DO was well separated by HPLC (Figures 1(a)–1(c)). The HPLC result indicated that DOP contained several main compounds including mannose (3.054%), ribose (0.030%), rhamnose (0.061%), glucuronic acid (0.028%), galacturonic acid (0.027%), glucose (1.296%), galactose (0.005%) and xylose (0.030%).

### 3.2. Glycemia and Body Weight

STZ-induced diabetic rats showed a significantly higher blood glucose level than the normal control group (*P* < 0.01, Table 1). Five weeks after the onset of diabetes, the blood glucose level did not change; however, the body weight of diabetic rats significantly decreased (*P* < 0.01, Table 1). After 5 weeks of treatment, DO did not exhibit a hypoglycaemic effect or inhibit the reduction of body weight (*P* > 0.05, Table 1). But in contrast, DO-treated rats were much more active with cleaner hair and brighter eyes than the nontreated diabetic rats.

### 3.3. Estimation of TG, TC, CREA, BUN, and GSH-PX

As shown in Table 2, the TC, TG, CREA, and BUN levels were significantly elevated in the diabetic control group when compared with the normal control group (*P* < 0.01 or *P* < 0.05). After 5 weeks of DO treatment, TC, TG, CREA, and BUN levels were significantly reduced in diabetic rats (*P* < 0.01). A significant decrease in serum GSH-PX levels was observed in the diabetic control group (*P* < 0.01), and DO treatment normalised GSH-PX levels in the diabetic rats (*P* < 0.01).

### 3.4. Determination of Electroretinographic Activity and Hypoalgesia

**Figure 2:**
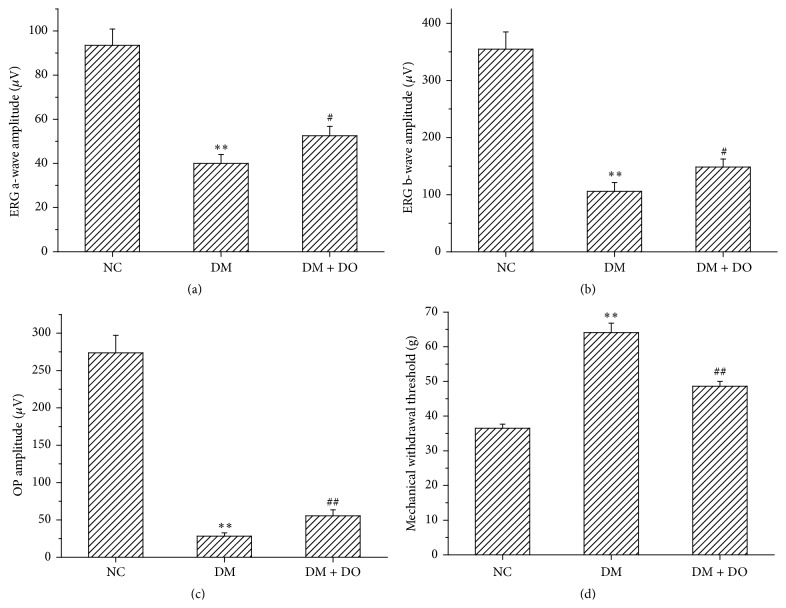
Effect of DO on scotopic ERGs and hypoalgesia. After 4 weeks of DO treatment, a significant increase in the amplitude of ERG a-wave (panel (a)), b-wave (panel (b)) and sum of OP (OPs; panel (c)) amplitude were observed compared with diabetic control. Panel (d) represented the punctuate mechanical withdrawal threshold from different treated group. Data are the mean ± S.E.M. (*n* = 8–10 animals per group). ^*∗∗*^ *P* < 0.01, compared with the normal control; ^##^ *P* < 0.01, ^#^ *P* < 0.05 compared with the diabetic control using ordinary ANOVA test.

The functional state of the retinas was analyzed by scotopic electroretinography. The average amplitudes of scotopic electroretinogram (ERG) a-waves and b-waves and OPs of each group are depicted in Figures 2(a)–2(c). These parameters were significantly reduced in the diabetic control group when compared with the normal controls (*P* < 0.01). DO treatment significantly increased the amplitudes of ERG a- and b-waves and OPs when compared with nontreated diabetic controls (*P* < 0.01 or *P* < 0.05; Figures 2(a)–2(c)).

The data in Figure 2(d) demonstrate that 5 weeks after the onset of diabetes, rats were hypoalgesic, as evidenced by the significant increase in punctate mechanical withdrawal thresholds when compared with the normal controls (*P* < 0.01). DO treatment prevented the hypoalgesia in diabetic rats (*P* < 0.01).

### 3.5. Histological Analysis of the Eyes, Hearts, Aortas, Kidneys, and Livers

**Figure 3:**
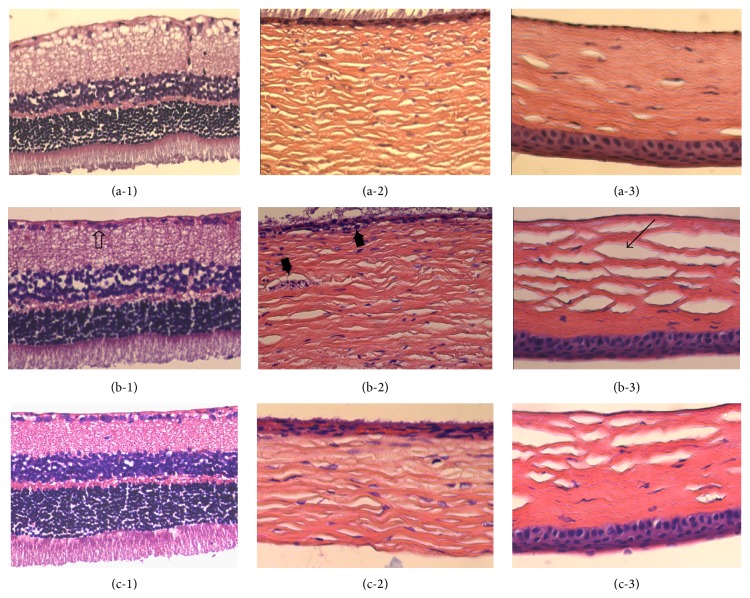
Effects of DO on eye damage in STZ-induced diabetic rats. Representative microscopic photographs of hearts stained with H&E (magnification ×400). Eye sections were obtained from the normal control group ((a-1)–(a-3)), untreated diabetic group ((b-1)–(b-3)), and DO-treated diabetic group ((c-1)–(c-3)). Atrophy of granular layer (*➩*); neovascularization and increasing density of endotheliocyte nuclei (*➨*); vessel dilation (→).

In nontreated diabetic rats, slight atrophy of the granular layer was observed when compared with the normal control group (Figure 3(b-1)). Retinal neovascularization and increasing density of endotheliocyte nuclei could be observed beyond the inner limiting membrane (Figure 3(b-2)). Obvious choroid small vessel dilation was also present in the eyes of diabetic rats (Figure 3(b-3)). In DO-treated diabetic rats, retinal vessel dilation was improved and did not show neovascularisation. No evident changes in the lens or corneas were observed among the groups.

**Figure 4:**
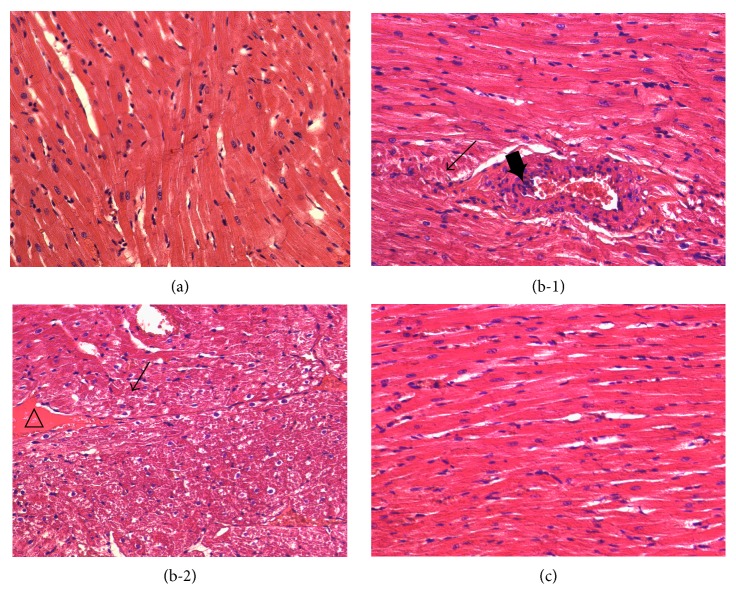
Effects of DO on heart damage in STZ-induced diabetic rats. Representative microscopic photographs of hearts stained with H&E (magnification ×400). Heart sections were obtained from the normal control group (a), untreated diabetic group ((b-1)-(b-2)), and DO-treated diabetic group (c). Cardiomyocyte hypertrophy and myofibril disorganization (→), endothelial hyperplasia (*➨*), and hyperaemia (△).

Five weeks after the onset of hyperglycemia, hearts from untreated diabetic rats displayed obvious cardiomyocyte hypertrophy, myofibril disorganization, small vessel hyperaemia, and endothelial hyperplasia (Figures 4(b-1) and 4(b-2)). In the DO-treated group, the cardiomyocyte hypertrophy and vessel hyperaemia were markedly attenuated. In addition, endothelial hyperplasia of small vessels was not found (Figure 4(c)).

**Figure 5:**
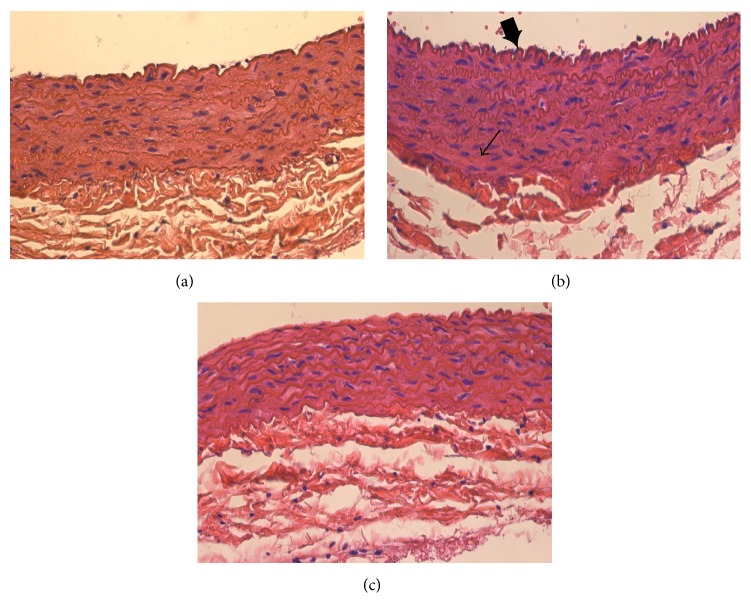
Effects of DO on aorta damage in STZ-induced diabetic rats. Representative microscopic photographs of aortas stained with H&E (magnification ×400). Aorta sections were obtained from the normal control group (a), untreated diabetic group (b), and DO-treated diabetic group (c). Smooth muscle cell hypertrophy (→), endothelial proliferation (*➨*).

In the diabetic rats, hyperglycemia caused swelling of the aortic smooth muscle and hyperplasia; furthermore, we found a thickening of the vascular wall and arterial endothelial cell proliferation (Figure 5(b)). These observations were significantly attenuated by treatment with DO (Figure 5(c)).

**Figure 6:**
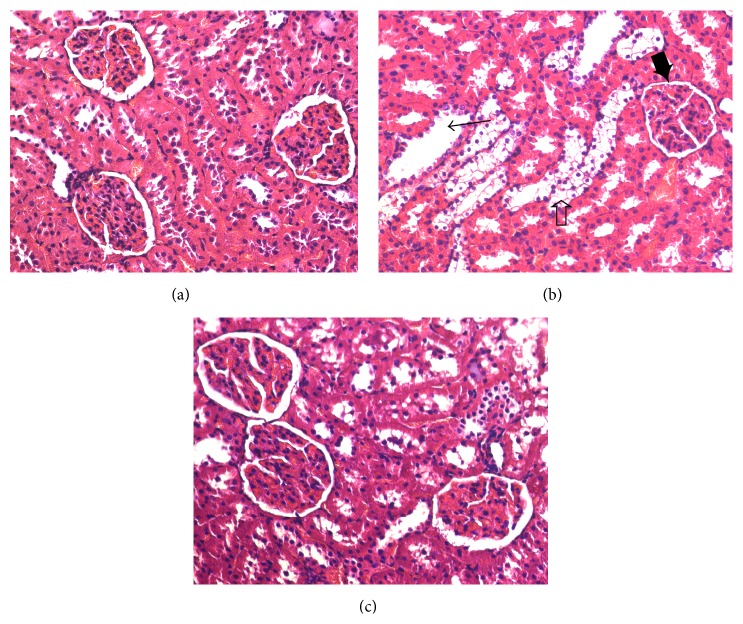
Effects of DO on kidney damage in STZ-induced diabetic rats. Representative microscopic photographs of kidneys stained with H&E (magnification ×400). Kidney sections were obtained from the normal control group (a), untreated diabetic group (b), and DO-treated diabetic group (c). Tubular dilation (→), glomerular space reduction (*➨*), and glycogen deposition (*➩*).

In the untreated diabetic rats, histopathological examination of the kidneys revealed changes in the renal tubules and glomeruli (Figure 6(b)). In the renal tubules, glycogen deposition in the tubular epithelium and tubular dilation were noted. The glomerular space was markedly reduced (Figure 6(b)). The renal damage in diabetic rats was largely attenuated by DO treatment (Figure 6(c)).

**Figure 7:**
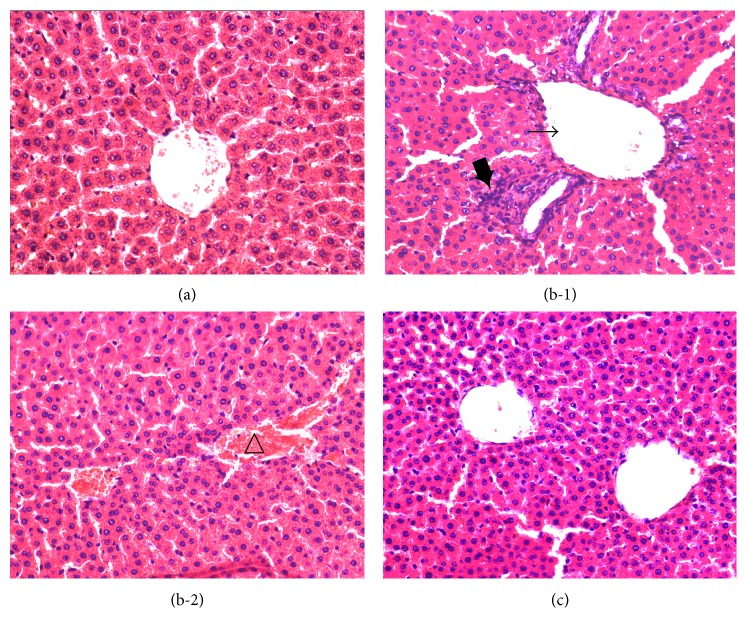
Effects of DO on liver damage in STZ-induced diabetic rats. Representative microscopic photographs of livers stained with H&E (magnification ×400). Liver sections were obtained from the normal control group (a), untreated diabetic group ((b-1)-(b-2)), and DO-treated diabetic group (c). Central vein dilation (→), lobular inflammation (*➨*), and hyperaemia (△).

In normal control rats, the fine hepatic lobule was clearly visible in the central vein, and the plates of hepatocytes and hepatic sinusoids radiated from the central vein (Figure 7(a)). In the STZ-induced diabetic rats, the hepatic lobule presented with a cloudy swelling of hepatocytes (Figure 7(b-1)). Obvious central vein dilation, hyperaemia, and mononuclear cell infiltration were also detected (Figures 7(b-1) and 7(b-2)). The liver of DO-treated diabetic rats exhibited better arranged hepatocytes without other obvious pathological changes (Figure 7(c)).

## 4. Discussion

Diabetes is associated with higher risks of death involving the circulatory system, respiratory system, digestive system, and genitourinary system [16]. Till now, there are rare reports about the medicine that can attenuate synchronously the damage on main organs of diabetics. In this study, we investigated the pathological changes in several vital organs and evaluate the protective effect of DO.

To follow the development of retinal damage, electroretinogram (ERG) and histological analyses were completed. Three ERG parameters were used to assess the functional integrity. The maximal amplitudes of a-wave and b-wave were used as indicators for photoreceptor function and the bipolar cells function, respectively. The sum of the oscillatory potentials was used to assess the function of the inner retina [17–19]. In Figures 1(a)–1(c), an ERG deficit was found 5 weeks after the induction of diabetes indicating that the functional integrity of the retina had been compromised. In support of the ERG findings, histopathological changes in the retina of diabetic rats also showed damage. Treatment with DO significantly attenuated the ERG deficits and pathological changes in the eyes of diabetic rats.

Neuropathy, characterised by weakness, sensory impairment and autonomic dysfunction, is one of the most pervasive symptoms in diabetic patients [20]. The data in Figure 2(d) suggest that hyperglycemia produces marked hypoalgesia. The quantifiable behavioural endpoint directly reflects a sensitisation of nociceptive processing and indicates the presence of punctuate mechanical hyperalgesia [11]. Our results indicate that treatment of diabetic rats with DO was effective in improving punctuate mechanical nociception.

Kidneys remove metabolic wastes, such as urea, uric acid, creatinine, and ions. The concentrations of metabolites can increase in the blood because of renal diseases or renal damage associated with uncontrolled diabetes mellitus [21]. In the present study, there was an elevation in urea nitrogen and creatinine in the diabetic group, suggestive of renal damage. These biochemical parameters correlated with the renal histological studies. We determined that STZ caused a significant damage to renal structures, including the glomeruli and tubules. However, in the DO-treated animals, levels of urea nitrogen and creatinine were significantly decreased. In addition, the administration of DO prevented these histological alterations.

Cardiovascular damage is another common complication of diabetes mellitus. The STZ-induced diabetic rat is an excellent model to examine the pathophysiology of diabetic cardiomyopathy [22]. Therefore, we employed this model to test the preventive effects of DO on cardiovascular damage. Diabetic cardiomyopathy is characterised by altered cardiac structure. In our current study, we observed a clear disorganization of myofibrils, myocyte hypertrophy, and vessels hyperaemia in the hearts of STZ-induced diabetic rats. Smooth muscle cell proliferation and endothelial proliferation were also evident in the diabetic rat aorta. Importantly, DO protected against these histopathological changes.

Diabetes mellitus is typically associated with an increase in plasma lipid levels. We also observed an increase in TG and TC levels in STZ-induced diabetic rats. Administration of DO for 5 weeks normalised the lipid profile of diabetic animals. These data indicate that DO can significantly improve the imbalance in lipid metabolism. Furthermore, DO attenuated hyperglycemia-induced liver damage, which may also help to improve the lipid metabolism in diabetes mellitus subjects.

Free radicals and associated reactive oxygen species have been implicated in diabetes as a cause for the development of some complications [23, 24]. It has been suggested that a variety of antioxidants that scavenge reactive oxygen species might improve hyperglycemia and prevent these diabetic complications. In our present study, we found that DO significantly increases the serum level of glutathione peroxidase in diabetic rats. Thus, we believe that the antioxidant activity of DO might play an important role in preventing diabetic complications. However, DO had no effect on blood glucose levels and bodyweight of diabetic rats. One of the reason might be administration period was not long enough. So we need to extend the administration period to further confirm the potential efficacy on blood glucose levels and study the underlying mechanism to prevent diabetic complications.

## 5. Conclusion

Currently, medical prevention and treatment of diabetic complications are mainly based on the optimised control of blood glucose. Our present study shows that treatment with DO clearly attenuates the early complications in STZ-induced diabetic rats, even though DO does not exhibit a hypoglycaemic effect. DO is nontoxic with no reported side effects, and it has been used for hundreds of years. Therefore, oral supplementation with DO could be an excellent supplement for the treatment of diabetic complications.

## Figures and Tables

**Table 1:** Effect of DO on body weight and blood glucose levels of diabetic rats.

Groups	Body weight (g)		Blood glucose (mM/L)	
	0 weeks	4 weeks	0 weeks	4 weeks
NC	315.0 ± 9.9	348.1 ± 8.5	5.4 ± 1.2	5.7 ± 1.5
DM	305.3 ± 8.2^*∗∗*^	230.8 ± 10.7^*∗∗*^	33.1 ± 0.8^*∗∗*^	31.9 ± 1.8^*∗∗*^
DM + DO	309.8 ± 12.9^*∗*^	235.7 ± 14.9^*∗∗*^	32.5 ± 1.4^*∗∗*^	30.3 ± 2.5^*∗∗*^

Data are expressed as mean ± S.E.M.; number of rats per group *n* = 8. ^*∗∗*^ *P* < 0.01, ^*∗*^ *P* < 0.05 compared with the normal control using ordinary ANOVA test.

**Table 2:** Effect of DO on the serum levels of TG, TC, CREA, and BUN in diabetic rats.

Groups	TG (mM/L)	TC (mM/L)	CREA (*μ*M/L)	BUN (mM/L)	GSH-PX (U/L)
NC	0.87 ± 0.30	1.25 ± 0.26	33.7 ± 9.1	9.83 ± 0.81	1780 ± 50
DM	1.90 ± 0.49^*∗∗*^	1.59 ± 0.16^*∗*^	105.4 ± 22.4^*∗∗*^	26.15 ± 1.53^*∗∗*^	786 ± 70^*∗∗*^
DM + DO	0.77 ± 0.29^##^	1.08 ± 0.10^##^	61.8 ± 12.8^##^	15.11 ± 1.87^##^	1488 ± 31^##^

Data are expressed as mean ± S.E.M.; number of rats per group *n* = 6–8. ^*∗∗*^ *P* < 0.01, ^*∗*^ *P* < 0.05 compared with the normal control; ^##^ *P* < 0.01 compared with the diabetic control using ordinary ANOVA test. TG: triglyceride; TC: total cholesterol; CREA: creatinine; BUN: urea nitrogen; GSH-PX: glutathione peroxidase.
